# Efficacy of Core Stability Exercises and Intrinsic Foot Training on Patients With Flat Foot: A Comparative Study

**DOI:** 10.7759/cureus.95659

**Published:** 2025-10-29

**Authors:** Jaykumar Soni, Saloni Sawant, Saiyam Shah

**Affiliations:** 1 Musculoskeletal and Sports, College of Physiotherapy, Sumandeep Vidyapeeth Deemed to Be University, Vadodara, IND

**Keywords:** core stability exercises, flatfoot, foot function index, intrinsic exercises, navicular height, normalized truncated navicular height, nprs (numeric pain rating scale), physiotherapy management, short foot exercises

## Abstract

Background

Flatfoot, or Pes planus, is a common condition characterized by the collapse of the medial longitudinal arch, leading to instability and balance issues. Strengthening intrinsic foot muscles through short-foot exercises is a widely used rehabilitation approach. Additionally, core stability exercises (CSE) play a crucial role in maintaining musculoskeletal stability, as the core and the foot are anatomically and functionally connected via the superficial back line. The objective of the study was to evaluate and compare the effectiveness of core stability exercises and intrinsic foot training in individuals with flatfoot.

Methods

A randomized controlled trial (RCT) was conducted with 80 participants diagnosed with flatfoot. They were randomly assigned to either the Core Stability Exercise (CSE) group (n=40) or the Intrinsic Foot Training (IFT) group (n=40). The intervention lasted for four weeks, with exercises performed three times per week on alternate days. The primary outcome measure was Normalized Truncated Navicular Height (NTNH), while the secondary outcome measure was the Foot Function Index Questionnaire (FFIQ). Baseline and post-intervention assessments were conducted to evaluate the effectiveness of the interventions.

Results

The results indicated significant within-group improvements in NTNH and FFIQ in both groups. In the CSE group, there were significant improvements in NTNH (Z=-5.604, p=0.000) and FFIQ (Z=-5.553, p=0.000). Similarly, the IFT group showed significant changes in NTNH (Z=-5.591, -5.577, p=0.000) and FFIQ (Z=-5.539, p=0.000). However, intergroup comparisons showed no significant differences in NTNH between the two groups (right leg: Z=-0.774, p=0.439; left leg: Z=-0.359, p=0.72). A significant difference in FFIQ (Z=-1.917, p=0.05) was observed, favoring the CSE group, indicating a better functional improvement compared to the IFT group.

Conclusion

In conclusion, both core stability and intrinsic foot training were effective in improving NTNH and foot function in individuals with flatfoot. However, the CSE group demonstrated a slightly greater improvement in foot function. These findings suggest that both interventions can be beneficial in managing flatfoot and should be incorporated into physiotherapy rehabilitation programs.

## Introduction

Pes planus is a complex deformity. It is a commonly observed condition [1]. Its prevalence is 13.6% (12.8% in males; 14.4% in females) [2]. The ankle is the furthest part of the lower extremity. It covers the minimum area of the human body. The function of the foot is to stand and support [3]. With weight bearing, the flexible medial arch flattens in spite of a high arch. So the disturbance can affect the functions of the foot. The intrinsic muscle provides surface while moving the body forward [4]. Weak muscles increase the risk of fasciitis, sprains, and injuries to other body parts. These muscles have an impact on the degree of foot pronation. Overuse injuries as patellofemoral-pain syndrome are been linked to increased risk of ankle. As a result, exercise in this area is important for maintaining the foot's core system [5].

Low back pain is due to disorders that affect the parts of the body. This maintains the medial arch of the foot. It is classified into flexible and rigid types. The arch remains flat in standing and sitting in a rigid over-pronated foot. While in a flexible pronated foot arch is flat only in standing, not in the sitting position [6]. The short-foot exercise is mostly performed in a flatfoot [7]. It maintains the arch of the foot and dynamic balance in standing. Continuous practice strengthens and maintains structure [8]. It includes exercises like great toe extension, towel curling, and reverse tandem walk [9].

The core muscles support the body when the arms and legs move. The term 'stability' is used instead of 'strength' as it is only one aspect needed [10]. Strengthening them can prevent injuries and musculoskeletal problems. These muscles connect the upper and lower limbs via the thoracolumbar fascia [11]. Multiple studies have focused on strengthening the body’s core. It involves exercises for the back and pelvis to build a strong core. These exercises build strength, endurance, and control for better movement [12]. A stable trunk is important for balance when shifting weight to one foot while moving. Core training provides strength, balance, and body control. 

The foot and upper body are linked together by the superficial back line. It connects the foot, calf, hamstrings, and lower back, through muscles and fascia. There is no study that core-muscle strengthening affects the plantar arch. Most studies focus on gender differences in foot muscle activity in flatfoot cases [13-17]. So, the goal is to check the link between flat foot, core stability and intrinsic-foot exercise.

## Materials and methods

**Figure 1:**
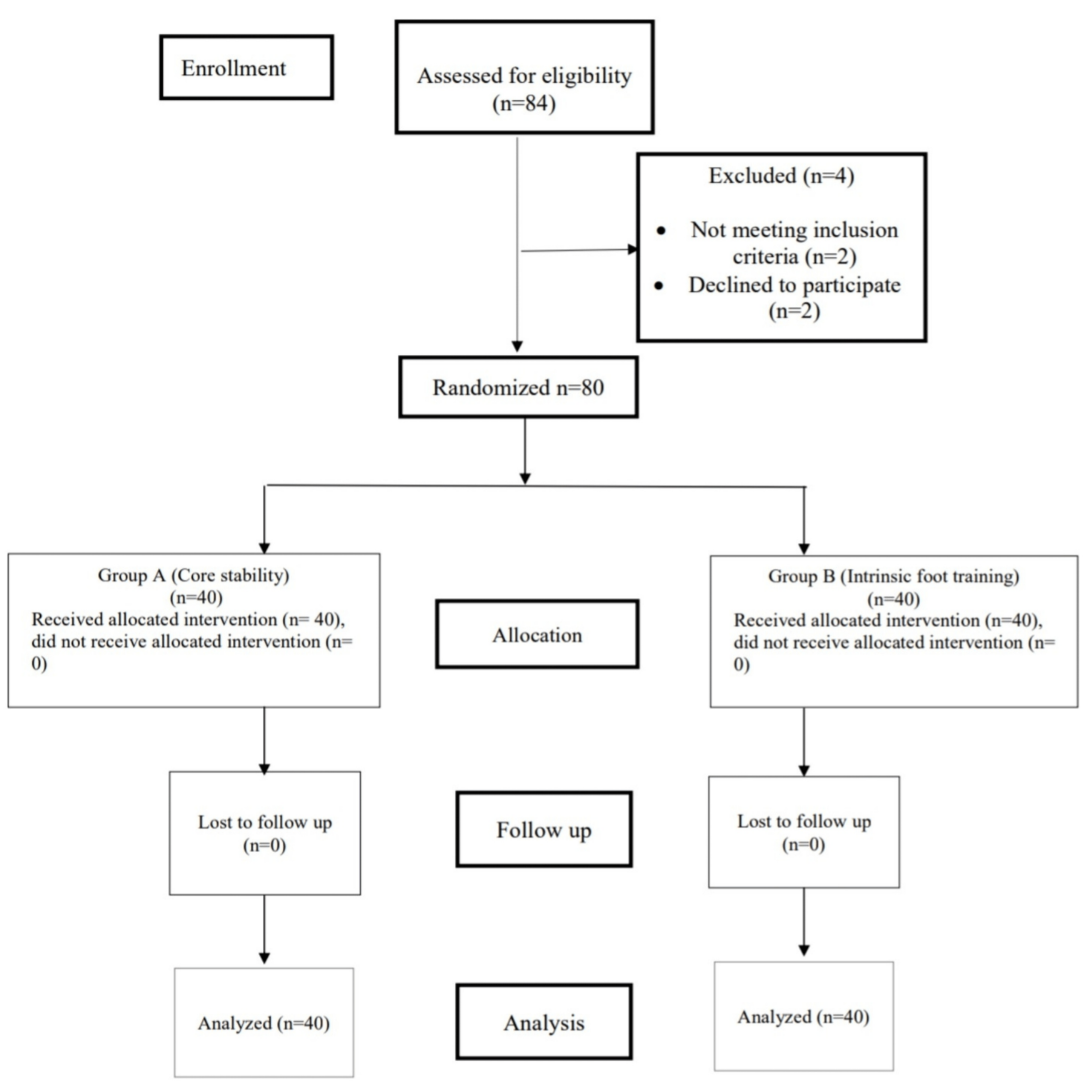
Consort flow chart

The randomized controlled trial was approved by Sumandeep Vidhyapeeth Institutional Ethical Committee (SVIEC/ON/PHYS/BNMPT22/OCH23/28) and registered for CTRI (CTRI/2023/11/060250). In the span of 12 months (1st August 2023 to 31st July 2024), 84 individuals were screened. However, four individuals were denied participation; thus, 80 individuals with flat feet were recruited (Figure 1).

The inclusion criteria were age 18 to 45 years, both male and female genders, and proficiency in Hindi, Gujarati, and English to understand and sign the consent and participants' information form. The subjects who had a history of lower limb soft tissue repair, unhealed fracture in the past year, any neurological conditions related to the foot, for example, diabetic neuropathy, deformity in the ankle and foot, and history of Spinal surgery were excluded. The sample size was calculated considering a 95% confidence interval and 5% level of significance, using the formula: 4pq/L2, where p = positive character, q = 1-p, L = allowable error in p (10 %).

Signed consent forms were obtained from participants, after which their demographic details and outcome measures: Normalized Truncated Navicular Height (NTNH) and Foot Function Index Questionnaire (FFIQ) were assessed. Both outcomes are open access. We calculated the NTNH by palpating and marking the navicular tuberosity with a blank index card aligned to the medial aspect of the foot while it was kept perpendicular to the floor. A mark was drawn on the index card at the level of the dot to indicate the navicular tuberosity. Later, the distance from the base of the index card to the level of the dot was measured in millimeters using a Vernier caliper to calculate the navicular height. Similarly, the navicular tuberosity was marked on the opposite lower limb, and its height was measured.

To obtain the participants’ footprints, we asked them to stand and press their feet on an ink pad and then place them on a piece of graph paper (Millimeter graph: 10 squares/centimeter) marked with their name, age, and sex. The graph papers were then left to air dry, and the participants were asked to place their feet on a microfiber sheet to absorb the excess ink and were seated comfortably and provided with tissue paper and isopropyl alcohol to clean their soles. After the footprints on the graph paper were dry, two parallel horizontal lines were drawn with a pencil. The first line was drawn at the level of the prominent aspect of the first metatarsophalangeal joint, and the second line was drawn at the most posterior part of the heel. To calculate the truncated foot length, the distance between these two parallel lines was measured in centimeters. The navicular height was converted to centimeters and divided by the truncated foot length with the help of a calculator to derive the NTNH. The arches of the patients’ feet were then classified as follows: ≤ 0.21 low; ≥ 0.22 normal; and ≥ 0.32 high [18-21].

The Foot Function Index (FFI) is a self-reported questionnaire. It includes three sections, a total of 23 questions related to pain (09), disability (09), and activity limitation (05). The total score is calculated as a percentage: FFI Score = Sum of item scores/Maximum possible score × 100. The interpretation of the score is as follows: 0-30 = lower disability, 31-60 = moderate disability, 61-100 = severe disability (poor function). The FFI has shown high reliability with intra-rater reliability reported as ICC = 0.87-0.92 and has demonstrated good validity across different patient populations with foot and ankle disorders [20,22]. The participants were randomly allocated to two different groups. Group A participants were treated with intrinsic foot muscles exercises, and Group B were treated with Core muscles strengthening. In both groups, participants received warm-up exercises and cool-down exercises (Tables 1, 2). The detailed protocol for each group is as follows: 

**Table 1:** Warm-up exercises for Group A and B

S.N.	Name of warm-up exercise	Repetition
1.	Marching at one place	10 sets each for 5 minutes
2.	Low intensity skips	
3.	Arm circles	
4.	Jumping Jacks	

**Table 2:** Cool-down exercises for Group A and B

S.N.	Name of cool-down exercise	Repetition
1.	Breathing exercise	Five sets of each exercise with 10 seconds hold
2.	Shavasana	
3.	Hamstring stretch	
4.	Calf stretch	

In Group A, participants received Core stability exercises as shown in Table 3. 

**Table 3:** Core stability exercises for Group A

Weeks	Draw in maneuver	Planks (Prone, left, right)	Crunches	Trunk Rotation with Weights	Bilateral Leg Lowering
1	20 seconds hold and 1 set	10 seconds hold 3 sets each	10 sets	10 sets each	10 seconds hold 5 sets each
2	30 seconds hold and 1 set	15 seconds hold 3 sets each	15 sets	15 sets each	15 seconds hold 5 sets each
3	30 seconds hold and 2 sets each	30 seconds hold 2 sets each	20 sets	20 sets each	15 seconds hold 5 sets each
4	30 seconds hold and 2 sets each	30 seconds hold 2 sets each	20 sets	20 sets each	15 seconds hold 5 sets each

 In Group B, participants received intrinsic foot training exercises as shown in Table 4.

**Table 4:** Intrinsic training exercises of foot for Group B

Weeks	Short foot exercise	Toes Spread-Out Exercise	Towel Curl Exercise	Great Toe Extension	Reverse Tandem Walk
1	Sitting position	Five repetitions of five sets with 5 minutes rest between each set			
2	Standing on both legs				
3	Standing on one Leg				
4	Standing on one Leg				

The data were analyzed using SPSS software version 26.0 (IBM Corp., Armonk, NY). The statistical tests used were Pearson, Mann-Whitney U, Wilcoxon signed-rank, and the Shapiro-Wilk test. 

## Results

A total of 80 patients were recruited for the study and were randomly allocated into two groups: Group A, Core Stability Training (n = 40), and Group B, Intrinsic Foot Training (n = 40). The demographic and baseline characteristics of participants, including gender distribution, occupation, and the type of footwear used, were assessed to ensure comparability between groups. Gender distribution showed a predominance of female participants in both groups, with Group A comprising 75% females and 25% males, while Group B included 85% females and 15% males (Table 5).

**Table 5:** Gender distribution in Group A and B

Gender	Group A	Group B
	Number (Percentage)	Number (Percentage)
Male	10 (25%)	6 (15%)
Female	30 (75%)	34 (85%)

Occupational analysis revealed a similar trend in both groups: Group A consisted of 25 workers and 15 students, whereas Group B comprised 24 workers and 16 students (Table 6).

**Table 6:** Occupation distribution between Group A and Group B

Occupation	Group A	Group B
Worker	25	24
Students	15	16

With regard to footwear habits, the majority of participants in Group A wore shoes (62.5%), followed by sandals (22.5%) and slippers (12.5%). In Group B, shoes were used by 40% of participants, sandals by 27.5%, and slippers by 7.5% (Table 7).

**Table 7:** Types of footwear

Type of footwear	Group A	Group B
Shoes	25	16
Sandals	9	11
Slippers	5	3

In group A, significant improvements were observed across all outcome measures: Nemeric Pain rating Scale (NPRS) pain score, Foot function Index (FFI), both right and left Navicular Heights (NH), Truncated foot length (L), Normalized truncated navicular height (NTNH) following the core stability exercise (Table 8).

**Table 8:** Intragroup comparison of NPRS, navicular height, truncated foot length (L), NTNH and FFI in Group A NPRS: Numeric Pain Rating Scale, NTNH: Normalized Truncated Navicular Height, FFI: Foot Function Index.

Outcome Measures		Pre	Post	Z Value	P Value	Interpretation
		Mean ± SD	Mean ± SD			
NPRS		3.95 ± 0.845	2.97 ± 0.831	-6.245	0.000	Statistically Significant
FFI		33.61 ± 4.61	24.88 ± 4.67	-5.553	0.000	Statistically Significant
NH	Right	3.225 ± 0.479	4.2 ± 0.464	-6.245	0.000	Statistically Significant
	Left	3.225 ± 0.479	4.2 ± 0.464	-6.245	0.000	Statistically Significant
L	Right	18.177 ± 0.809	18.295 ± 0.873	-6.091	0.000	Statistically Significant
	Left	18.102 ± 0.781	18.21 ± 0.76	-6.091	0.000	Statistically Significant
NTNH	Right	0.172 ± 0.023	0.229 ± 0.022	-5.604	0.000	Statistically Significant
	Left	0.177 ± 0.023	0.230 ± 0.0216	-5.617	0.000	Statistically Significant

In group B, significant improvements were observed across all outcome measures: pain score (NPRS), Foot function Index (FFI), both right and left Navicular Heights (NH), Truncated foot length (L), Normalized truncated navicular height (NTNH) following the intrinsic foot muscle training (Table 9).

**Table 9:** Intragroup comparison of NPRS, Navicular height, Truncated foot length (L), NTNH and FFI in Group B NPRS: Numeric Pain Rating Scale, NTNH: Normalized Truncated Navicular Height, FFI: Foot Function Index.

Outcome Measures		Pre	Post	Z Value	P Value	Interpretation
		Mean ± SD	Mean ± SD			
NPRS		3.625 ± 1.054	2.65 ± 0.948	-5.794	0.000	Significant
FFI		30.012 ± 5.114	22.1 ± 4.259	-5.539	0.000	Statistically significant
NH	Right	3.15 ± 0.426	4.102 ± 0.378	-6.172	0.000	Statistically significant
	Left	3.15 ± 0.426	4.102 ± 0.378	-6.172	0.000	Statistically significant
L	Right	18.187 ± 0.803	18.305 ± 0.867	-6.091	0.000	Statistically significant
	Left	18.112 ± 0.776	18.217 ± 0.757	-6.008	0.000	Statistically significant
NTNH	Right	0.173 ± 0.024	0.225 ± 0.02	-5.591	0.000	Statistically significant
	Left	0.174 ± 0.025	0.225 ± 0.021	-5.577	0.000	Statistically significant

The comparisons between Groups A and B revealed no statistically significant differences in the outcome measures except for FFI (Table 10). For pain (NPRS), both groups achieved an identical mean reduction of -0.975, with no significant difference (Z = -0.028, p = 0.978). In Navicular Height (NH), Truncated Foot Length (L), and NTNH Improvements were similar across groups, with no significant intergroup differences (p > 0.05). However, in FFI, a significant difference was observed, with Group A showing a slightly greater mean improvement (-8.23 ± 2.34) compared to Group B (-7.68 ± 2.04) (Table 10).

**Table 10:** Intergroup comparison of NPRS, Navicular height, Truncated foot length (L), NTNH, and FFI between Group A and Group B NPRS: Numeric Pain Rating Scale, NTNH: Normalized Truncated Navicular Height, FFI: Foot Function Index.

Outcome Measures		Group A	Group B	Z Value	P Value	Interpretation
		Mean ± SD	Mean ± SD			
NPRS		-0.975±0.156	-0.975± 0.417	-0.028	0.978	Statistically not significant
FFI		-8.227±2.34	-7.675±2.041	-1.917	0.05	Statistically Significant
NH	Right	0.975±0.156	0.952±0.207	-0.57	0.569	Statistically not significant
	Left	0.975±0.156	0.952±0.207	-0.57	0.569	Statistically not significant
L	Right	0.1175±0.142	0.117±0.142	0	1	Statistically not significant
	Left	0.1075±0.081	0.105±0.083	-0.383	0.702	Statistically not significant
NTNH	Right	0.052±0.0089	0.05±0.011	-0.774	0.439	Statistically not significant
	Left	0.052±0.0087	0.051±0.0122	-0.359	0.72	Statistically not significant

## Discussion

The objective of the study was to examine the effects of core stability and intrinsic foot exercise in individuals. This type of training targets the smaller muscles within the foot that help maintain the arch of the foot and compares the effect of the two exercises. Till now, there has been no trial studied to see the role of core-stability exercises and intrinsic-foot training in patients with pes planus. However, one study compared core stability exercises with intrinsic foot training, focusing on outcomes like the Foot Posture Index and weight distribution during standing [18].

The study measured gender distribution across two groups, in which female participants were the highest in both groups compared to male participants. Occupational categories were also a key focus of this study. The groups had more labourers and fewer students. Another baseline characteristic was the type of footwear used by participants. Khamooshi et al. (2016) found that most people aged 15 to 35 years wear shoes [19]. Participants reported wearing shoes more often than slippers [20]. Similarly, Aboelnasr et al. (2019) found a similar trend in participants [21]. Budiman-Mak and Conrad (2019) found that the use of shoes and other footwear without high heels was required due to their job and safety [22].

It was found that hard-soled shoes reduce friction, thereby reducing fall risk. Most participants were familiar with their shoe size and less informed about it. Furthermore, many individuals prefer fashion over comfort, often wearing footwear that compromises foot health [23-25]. Branthwaite et al. (2014) discovered that footwear differences between groups were connected to age and foot pain [26]. Old women were more aware of the type of footwear worn, foot pain, and in shoe selection. But this study had limitations in the type of sole chosen and the material used. Participants from both groups found that low and flat shoes were less comfortable [26].

In this study, the baseline characteristics of participants are gender proportions, occupational roles, and footwear preferences. This study found that in Group A, the intragroup comparison of parameters used, including truncated foot length (L), NTNH, FFIQ, and NPRS, shows a significant difference in all parameters. Sedaghati et al. (2023) [20] and Hill et al. (2011) [27] found that exercises of the abdominal muscles, particularly core muscle strengthening, are necessary for the transfer of forces throughout the body and support the back region.

Branthwaite et al. (2014) concluded that exercises that focus on the core help improve balance during movement of the body. This is because the core functions as the body’s central stabilizing unit, providing postural alignment and stability when movements are initiated from the extremities [26]. Core exercises help in building strength and control in the body. They also support the spine and improve posture. When these muscles are strong, they prevent other injuries. So the activities like sports are easier and safer [23,28]. Core transfers all the movements from the upper and lower limbs of the body. So, a strong core improves overall body performance. A study by Aggarwal et al. (2010) concluded that a weak core can be a risk factor for lower limb injuries [29]. Hill et al. (2011) observed that an eight-week training program improved foot deformities in young females [27].

This study reveals that in Group B, the intragroup comparison showed significant improvements across all measures. Pabón-Carrasco et al. (2020) showed results that short-foot exercises minimize excessive foot pronation, strengthen intrinsic foot muscles, and improve arch stability [30]. These exercises evenly distribute the body weight and reduce strain on the foot. This prevents foot problems. The medial arch of the foot is formed with an increase in navicular height. Increased navicular height is associated with fewer balance problems and improved quality of life. The amount of pain is also reduced by doing these exercises. Aggarwal et al. (2019) found that foot exercises, when combined with modalities/NMES, had shown positive improvement [29].

The findings of this study proved that the comparison between Group A and Group B showed no visual changes. The abdominal muscles connect the upper and lower body together by a line that runs through the thorax and lumbar [10,28]. Research suggests that a weak core can lead to lower extremity injuries and over-foot-pronation. These findings suggest lower limb movements are affected. So exercises related to the core are considered important for the foot to prevent the collapse of the medial arch.

The arch of the foot collapses due to over-pronation of the foot. The intrinsic muscles of the foot provide support and maintain the arch. Research shows that strengthening these muscles helps to strengthen the arch [27]. Khisty et al. (2020) showed that the Short-Foot Exercise program was more beneficial for the medial arch [7]. This study showed a connection between footwear type and NPRS, with no changes. According to Pabón-Carrasco et al. (2020) women had more pain on back of foot. This was the only site of pain seen in women. Being overweight also causes foot pain in men and women. Only 2% of men reported poor-quality shoe pain. In men, footwear such as shoes does not play an important role in foot pain. But for women, wearing heels or uncomfortable shoes causes pain. So, women should think before buying footwear to reduce pain in the back of the foot and perform exercise [30].

Due to the higher proportion of female participants and the absence of long-term follow-up, the generalizability of results is limited. Future studies with larger, more diverse samples and extended intervention periods are recommended to establish long-term efficacy and to explore the combined effects of core and intrinsic foot training for managing pes planus.

## Conclusions

The present study concluded that both core stability exercises and intrinsic foot muscle training are effective in enhancing medial longitudinal arch height, foot function, and pain reduction in individuals with flat feet. Strengthening the core muscles contributes to improved postural control and stability between the upper and lower limbs, while intrinsic foot exercises directly target the small stabilising muscles of the foot, promoting better arch alignment and balance. Although both interventions demonstrated comparable overall improvements, participants who performed intrinsic foot muscle exercises showed greater enhancement in the Foot Function Index (FFI), indicating superior functional benefits in daily activities. These findings suggest that incorporating both exercise approaches in rehabilitation programs can yield comprehensive improvements in foot bio-mechanics and lower limb function.
